# The *Ganoderma lucidum* and *Rosa roxburghii* Tratt Formulation Prevents Depressive-like Behaviors in Mice by Modulating Tryptophan Metabolism via the Gut–Brain Axis and Upregulating the BDNF/TrkB/PI3K/AKT Pathway

**DOI:** 10.3390/foods15091535

**Published:** 2026-04-28

**Authors:** Fangling Feng, Shuo Zhang, Chencen Lai, Zhiyu Chen, Jian Zhang, Jiuming He, Min Zhang, Pengjiao Wang, Xiuli Gao

**Affiliations:** 1 State Key Laboratory of Discovery and Utilization of Functional Components in Traditional Chinese Medicine, School of Pharmaceutical Sciences, Guizhou Medical University, Guiyang 561113, China; fanglingfeng129@163.com (F.F.); zhangshuo@gmc.edu.cn (S.Z.); 2022010030128@stu.gmc.edu.cn (C.L.); chenzhiyu@stu.gmc.edu.cn (Z.C.); jianzhang0808@163.com (J.Z.); minzhang@gmc.edu.cn (M.Z.); 2 Engineering Research Center of Microbiology and Biochemical Pharmaceutical, Guizhou Provincial Department of Education, Guiyang 561113, China; 3 Experimental Animal Center, Guizhou Medical University, Guiyang 561113, China; 4 State Key Laboratory of Bioactive Substance and Function of Natural Medicines, Institute of Materia Medica, Chinese Academy of Medical Sciences and Peking Union Medical College, Beijing 100050, China; hejiuming@imm.ac.cn; 5 Guizhou Provincial Engineering Research Center of Food Nutrition and Health, Guizhou Medical University, Guiyang 561113, China

**Keywords:** depression, gut–brain axis, *Ganoderma lucidum*, *Rosa roxburghii* tratt, tryptophan metabolism, 5-HT, BDNF

## Abstract

Depression is a common mental disorder that substantially impairs patients’ daily life and work. To identify natural and safe preventive options, we investigated the preventive effect and underlying mechanism of the *Ganoderma lucidum* and *Rosa roxburghii* Tratt formula (GLRRTF) on depression. A total of 72 chemical components in GLRRTF were identified by UHPLC-ESI-Q-Exactive Plus Orbitrap-MS Analysis. GLRRTF (containing 400 mg/kg of *G. lucidum* extract and 800 mg/kg of *R. roxburghii* extract per day), administered as a 1-week preventive intervention followed by 4 weeks of co-administration with chronic unpredictable mild stress, prevented the development of depression-like behaviors in male C57BL/6J mice and reduced neuronal damage in the hippocampus. Airflow-assisted desorption electrospray ionization mass spectrometry imaging and enzyme-linked immunosorbent assays showed that GLRRTF corrected abnormalities in neurotransmitter levels. The 16S rRNA sequencing indicated that GLRRTF restored dysbiosis of the gut microbiota. Metabolomic profiling revealed that GLRRTF increased the level of tryptophan and promoted tryptophan metabolism towards the 5-HT and indole pathways in feces and the brain. Western blot demonstrated that GLRRTF increased 5-HT production from tryptophan in the brain by regulating tryptophan hydroxylase 2 and DOPA decarboxylase. GLRRTF activated the PI3K/AKT pathway by regulating brain-derived neurotrophic factor and its receptor tropomyosin receptor kinase B. This research provides a comprehensive mechanistic understanding of GLRRTF’s preventive effect against depression, highlighting its potential as a novel, safe, and preventive functional food formulation.

## 1. Introduction

Depression is a prevalent mental disorder that imposes a substantial burden on individuals, families, and society [1]. While its etiology involves complex neurobiological mechanisms, including monoamine neurotransmitter dysregulation, neuroinflammation, impaired neuroplasticity, and gut–brain axis dysfunction [2], current pharmacological treatments are associated with limited efficacy in a significant proportion of patients and adverse effects such as nausea, sexual dysfunction, and insomnia [3]. These limitations have prompted growing interest in identifying safe, food-based interventions for mental health maintenance. Natural bioactive compounds derived from edible sources—including polyphenols, polysaccharides, peptides, flavonoids, terpenes, and saponins [4]—have attracted considerable attention for their ability to modulate gut microbiota composition, regulate neurotransmitter systems, and promote neurotrophic factor signaling [5]. Notably, accumulating evidence suggests that dietary components can enhance brain-derived neurotrophic factor (BDNF) expression and activate its downstream tropomyosin receptor kinase B (TrkB) signaling pathways, which are critical for synaptic plasticity and stress resilience [6]. Within this context, functional foods and food-derived formulations represent a promising avenue for developing safe and accessible strategies to support mental health, particularly for individuals who experience inadequate responses or adverse effects to conventional antidepressant therapies.

The gut–brain axis (GBA) serves as a bidirectional communication network connecting the gastrointestinal tract and the central nervous system, providing a mechanistic link through which dietary components and gut microbiota influence brain function and behavior [7]. Dysbiosis has been implicated in the pathophysiology of depression through multiple pathways, including immune activation, inflammation, hypothalamic–pituitary–adrenal (HPA) axis modulation, and regulation of tryptophan metabolism [8]. Tryptophan is an essential amino acid derived from dietary sources [9], and its metabolism is critically governed by the gut microbiota through three major pathways: the kynurenine, indole, and 5-hydroxytryptamine (5-HT/serotonin) pathways [10]. The microbes in the gut have the ability to actively regulate the level of 5-HT by secreting some metabolites and also modifying the kynurenine pathway [5]. Depression’s pathophysiological processes are associated with BDNF, which is crucial for neuroplasticity and the survival of neurons [11]. BDNF has been extensively studied in the context of the gut microbiota [12]. The various pathways through which the gut–brain axis can regulate BDNF expression and function include direct regulation by microbe-derived metabolites [13], immune–inflammatory signals [14], and modulation of BDNF due to the regulation of 5-HT [15]. The TrkB receptor, activated by BDNF, initiates three independent signaling cascades, one of which is the phosphatidylinositol 3-kinase/protein kinase B (PI3K/AKT) signaling pathway associated with synaptic plasticity [16]. Collectively, these interconnected pathways highlight the potential for food-derived bioactive compounds to promote mental health by modulating gut microbiota composition and leveraging the gut–brain axis.

The formulation of *Ganoderma lucidum* and *Rosa roxburghii* Tratt originates from a traditional tea beverage of the Dong ethnic group in Guizhou, China. *G. lucidum* has long been consumed as an edible fungus and functional food ingredient in Asia, valued for its health-promoting properties [17]. Its fruiting bodies, mycelium, and spores contain various bioactive components, including polysaccharides and triterpenoids [18]. Previous studies have reported that *G. lucidum* mycelium extract [19], spore extract [20], polysaccharides [21], and triterpenoids [22] exert beneficial effects on mood regulation in preclinical models. *R. roxburghii* is a nutrient-dense fruit rich in vitamin C, polysaccharides, phenols, triterpenoids, organic acids, and superoxide dismutase [23]. It exhibits multiple health benefits, including immunomodulatory, antioxidant, anti-aging, and anti-fatigue activities [24]. Notably, *R. roxburghii* fermented juice can improve CUMS-induced depression-like behaviors [25], its constituent Kaji-ichigoside F1(KF1) has been reported to exert neuroprotective [26] and rapid antidepressant effects [27], while its polysaccharides protect neural stem cells from glutamate-induced damage and modulate the gut microbiota [28].

The C57BL/6J mouse is commonly used in depression studies because its behavioral and neurobiological responses to stress are well characterized [29]. The chronic unpredictable mild stress (CUMS) paradigm is a well-established model that reliably induces depression-like behaviors—such as anhedonia, behavioral despair, and anxiety—through repeated exposure to mild stressors [30]. It also allows investigation of the gut–brain axis and tryptophan metabolism, pathways increasingly implicated in stress-related depression in humans [31].

Based on the health-promoting properties of both ingredients, this study evaluated the preventive effects of the *G. lucidum* and *R. roxburghii* formulation (GLRRTF) on stress-induced depressive-like behaviors. Using a CUMS mouse model, we examined its effects on gut microbiota, tryptophan metabolism, and the BDNF/TrkB/PI3K/AKT pathway. Through multi-omics and molecular validation, we aim to provide mechanistic insights into GLRRTF as a potential functional food formulation for mental health promotion.

## 2. Materials and Methods

### 2.1. Materials

The dried fruiting bodies of *Ganoderma lucidum* were purchased from Xubo Medicinal Herbs Store (Huaxi Avenue, Nanming District, Guiyang, Guizhou, China), and the fresh fruits of *Rosa roxburghii* Tratt were purchased from Guizhou Chaoshangwei Trading Co., Ltd. (Guiyang, Guizhou, China). Both materials were authenticated by Prof. Qing-de Long (Guizhou Medical University) based on morphological characteristics. Fluoxetine (F189157-1g) was purchased from Shanghai Aladdin Biochemical Technology Co., Ltd. (Shanghai, China). The enzyme-linked immunosorbent assay (ELISA) kits of 5-HT, dopamine (DA), norepinephrine (NE), and acetylcholine (Ach) were acquired from Fankewei Co., Ltd. (Shanghai, China). Ultra-high-performance liquid chromatography reagents were obtained from Merck (KGaA, Darmstadt, Germany) while others were of analytical grade. The primary antibodies included tryptophan hydroxylase 2 (TPH2) antibody (HA722195), DOPA decarboxylase (DDC) antibody (ET1704-94), BDNF antibody (ET1606-42), TrkB antibody (ET7111-38), Phospho-PI3K p85 (Y467) + PI3K p55 (Y199) (p-PI3K) antibody (HA721672), PI3Kinase p85 alpha (PI3K) antibody (ET1608-70), Phospho-AKT (S473) (p-AKT) antibody (ET1607-73), AKT1/2/3 (AKT) antibody (ET1609-51) and glyceraldehyde-3-phosphate dehydrogenase (GAPDH) antibody (60004-1-lg). Except for GAPDH which is from Proteintech Group, Inc. (Wuhan, Hubei, China), all other antibodies are from HUABIO (Hangzhou, Zhejiang, China).

### 2.2. Preparation of G. lucidum and R. roxburghii Extracts

The crude *G. lucidum* was washed, drained, oven-dried at 55 °C for 24 h (to <12% moisture), and ground to a coarse powder. Ultrapure water was added eight times the weight of the powder, which was decocted twice under boiling conditions for 3 h each time. The decoctions were combined, filtered through 200-mesh cloth, and insolubles were removed by centrifugation at 4000 rpm for 15 min at room temperature. The solution was then concentrated by rotary evaporator (RE-52A, Shanghai Yarong Biochemical Instrument Factory, Shanghai, China) (−0.08 Mpa, 75 °C) to final volume 50 mL. The cut fresh *R. roxburghii* pieces were decocted in water, and the following processing was conducted according to *G. lucidum*. A hundred grams of fresh *R. roxburghii* were dried at 65 °C to give 15.3 g of dried fruit. The dosing for the other trials was based on the weight of the dried *R. roxburghii*.

### 2.3. UHPLC-ESI-Q-Exactive Plus Orbitrap-MS Analysis

We ran the samples on a Thermo Vanquish Horizon UHPLC system (Thermo Fisher Scientific, San Jose, CA, USA) with a Hypersil Gold C18 column (2.1 × 100 mm, 1.9 μm) to get a first look at the chemical compounds in GLRRTF. The mobile phases were A, 0.1% formic acid in water; B, 0.1% formic acid in acetonitrile. The gradient went like this: 0–3 min, 2% B; 3–23 min, B increased linearly to 98%; 23–26 min, stayed at 98%; 26–26.1 min, dropped back to 2%; 26.1–28 min, kept at 2%. Injection volume was 2 μL, flow rate 0.3 mL/min, and column temperature 40 °C. For MS, we used an ESI-Q-Exactive Plus Orbitrap system in both positive and negative ion modes. The main parameters were spray voltage ± 3.5/2.5 kV, vaporizer temperature 350 °C, capillary temperature 320 °C. We ran a full MS/dd-MS^2^ scan from *m*/*z* 100 to 1500, with MS^1^ resolution at 70,000 and MS/MS at 17,500. The stepped NCEs were 20, 40, and 60. Raw data were put into Compound Discoverer 3.2, and we accepted a mass error within 5 ppm. Data processing used accurate mass and MS/MS fragments from Xcalibur 4.3. We also crosschecked with standard spectra and fragment data from *m*/*z* Cloud, *m*/*z* Vault, Masslist, MoNA, ChemSpider, and published papers. The mzLogic algorithm helped with confidence. Finally, we used Mass Frontier 7.0 to help assign fragments and confirm the structures of the active compounds in GLRRTF.

### 2.4. Animal Experiments

#### 2.4.1. Establishment of Animal Model and Drug Administration

We developed a depression animal model by applying the combination of chronic unpredictable mild stress (CUMS) and isolation rearing in mice. The study utilized 48 male C57BL/6 J mice aged 6–8 weeks, with a weight range of 18–22 g. The mice were obtained from Sibefu (Beijing) Biotechnology Co., Ltd. (Beijing, China). were specific pathogen-free (SPF) as reported by the supplier, were wild-type with no genetic modifications, and had no prior experimental procedures. They were kept in an environment with a temperature of 23 ± 1 °C and humidity of 50 ± 10%, following a 12 h light/dark cycle. The animals could freely access both food and water. The trials involving animals adhered strictly to the Guide for Care and Use of Laboratory Animals. The Animal Care and Use Committee of Guizhou Medical University approved these experiments (License No. SCXK (Jing) 2024-0001, Animal Experiment Ethics Review No. 2403733). The mice were given a week to adapt to the environment. Subsequently, mice were randomly assigned to groups using a computer-generated random number sequence. A negative control group consisted of non-stress (ordinary) mice, while treatment groups included the CUMS group (CUMS + ultrapure water). Each of these groups had 12 mice. The other treatment groups were the GLRRTF (CUMS + GLRRTF) and fluoxetine (FLX) positive control group (CUMS + FLX). The GLRRTF extract was prepared by mixing the dried extracts of *G. lucidum* and *R. roxburghii* at a fixed ratio of 1:2 (*w*/*w*), based on our registered product (Dongxiu Lijing *Ganoderma lucidum* and *Rosa roxburghii* Granules, No. G20240113). The combined extract was suspended in ultrapure water. The *G. lucidum* extract was administered at 400 mg/kg per day, and the *R. roxburghii* extract at 800 mg/kg per day. The human *G. lucidum* dose (3000 mg/70 kg ≈ 43 mg/kg per day) was converted to mouse dose using factor 9.1 [32]: ≈390 mg/kg, rounded to 400 mg/kg. The 1:2 ratio then determined the *R. roxburghii* dose of 800 mg/kg. The FLX group received fluoxetine at 20 mg/kg per day, a dose widely reported to be effective in the CUMS mouse model [33]. All were given orally at 0.1 mL/10 g body weight. Both agents were delivered via the intragastric route daily. Both control and CUMS groups were administered equal volumes of ultrapure water (0.3 mL). After one week of preventive administration, the mice received daily GLRRTF (or ultrapure water for controls) concurrently with a 4-week CUMS protocol. The CUMS protocol was designed based on the established literature [34]. Thirteen different mild stressors were used that included food and water deprivation, empty cage, wet bedding, restraint, tilted cage, noise, heat exposure, swimming in cold water, overnight stroboscopic light, shaken cage, night lighting and light-off in the daytime. Daily stressors were applied from 09:00 to 22:00 and not on 24 h stressors. In order to avoid the predictability of stressful events occurring at a certain time, mice were separated from each other, and two stressors were randomly introduced each day. At just one hour after administering the drug, stressors were applied. Behavioral tests were done in a soundproof room after CUMS modeling with behaviors recorded. No humane endpoints were applied as all animals completed the study without meeting predefined exclusion criteria. All animals completed the study without signs of severe distress or illness. At the end of the experiment, mice were euthanized by cervical dislocation under isoflurane anesthesia. Investigator administering CUMS and treatments was aware of allocation (unavoidable). Data analysis was performed by an investigator who was not involved in behavioral testing and was unaware of group allocation until the final analysis.

#### 2.4.2. Sucrose Preference Test (SPT)

Mice were first given two bottles of 1% sucrose water for 24 h. Then, one bottle was replaced with ultrapure water for another 24 h. After this 48 h habituation [35], mice were deprived of food and water for 24 h. Each mouse was singly housed and then received one bottle of 1% sucrose water and one bottle of ultrapure water. After 24 h, the bottles were removed and weighed. Sucrose preference (%) = (sucrose intake/total fluid intake) × 100%. Sucrose preference was the primary outcome. Sample size (*n* = 12 per group) was based on previous CUMS studies, as no formal power calculation was performed.

#### 2.4.3. Open Field Test (OFT)

The apparatus was a square box (40 × 40 × 40 cm) under dim light and quiet conditions. Mice were brought to the testing room and left in their home cages for 30 min before the test. No prior exposure to the box was given. Each mouse was placed in a fixed corner and allowed to explore for 5 min. Behavior was recorded by a camera and analyzed with ANY maze software 7.3.3. The time spent in the central area (about 1/3 of the floor) and total distance traveled were measured [36].

#### 2.4.4. Forced Swimming Test (FST)

A clear circular container (12 cm diameter, 25 cm deep) was filled with water (23–25 °C). One day before the test, each mouse was placed in the water for 15 min (pretest) [37]. On the test day, mice were placed in the testing room for 30 min, then put into the container for 6 min. A camera recorded the session, and immobility time during the last 4 min was scored.

#### 2.4.5. Tail Suspension Test (TST)

A strip of tape was attached to the tail at about one third from the tip, and the mouse was hung upside down from a rod, with the head 15 cm above the floor. Mice were acclimated to the testing room for 30 min before the test. The test lasted 6 min, and immobility time during the last 4 min was analyzed. No prior training was given [38].

#### 2.4.6. Elevated Plus Maze Test (EPMT)

The maze was 100 cm above the floor, with two open arms (50 × 10 cm), two closed arms (50 × 10 × 40 cm), and a central platform (10 × 10 cm). Mice were brought to the testing room for 45 min before the test [39]. There was no prior exposure to the maze. Each mouse was placed at the center and allowed to explore for 5 min. The time spent in and entries into open arms were recorded. A valid entry required all four paws to cross the line into an arm.

#### 2.4.7. FLX Group

The FLX group was included as a positive control in the behavioral tests (SPT, OFT, FST, TST, EPMT), histopathological staining, ELISA measurements of neurotransmitter levels, and Western blot analyses. Due to budget limitations, the FLX group was not included in the multi-omics assays (brain mass spectrometry imaging, 16S rRNA sequencing, and metabolomics of fecal, serum, and brain samples).

### 2.5. Histopathological Staining and Biochemical Detection

5-HT, DA, Ach, and NE levels in mouse brain tissue were measured using commercial ELISA kits following the manufacturer’s protocols. For histology, brains were fixed in 4% paraformaldehyde for 24 h, embedded in paraffin, and sectioned at 5 μm. H & E staining was performed as described [40]: hematoxylin 5 min → water rinse 5 min → 1% acid alcohol differentiation 2 s → 1% ammonia water bluing 30 s → eosin 30 s. Nissl staining was performed as described [41]: 0.1% cresyl violet at 37 °C for 20 min. Stained sections were examined under a light microscope (Nikon Corporation, Tokyo, Japan) (200×). All steps were carried out at room temperature unless specified. Neuronal apoptosis, pyknosis, and necrosis were identified under light microscopy following published criteria [42,43]. Apoptotic cells showed shrunken bodies, condensed nuclei, and dark cytoplasm (eosinophilic on H&E, basophilic on Nissl). Pyknotic nuclei were shrunken, dark, and lacked fine chromatin detail. Necrotic cells had hyper eosinophilic cytoplasm (H & E) or lost Nissl substance (Nissl), often with nuclear pyknosis or karyolysis. We distinguished necrosis from dark neuron artifacts by looking for various stages of necrosis or nearby neuropil edema and inflammation.

### 2.6. Airflow-Assisted Desorption Electrospray Ionization Mass Spectrometry Imaging

Brain tissues from three mice per group were collected and rapidly frozen at −80 °C for subsequent analysis. Coronal sections from the left hemisphere and sagittal sections from the right hemisphere of the mice brain were prepared. Brain tissues were sectioned into 12 μm slices at −22 °C using a Leica CM3050S cryostat (Leica Microsystems, Wetzlar, Germany) and mounted on glass slides. Adjacent tissue sections underwent coronal H & E staining to evaluate tissue morphology and confirm specific brain regions. Before the analysis, all tissue sections were stored in dry vacuum desiccators for approximately 90 min. The Q-OT-qIT hybrid mass spectrometer (Thermo Fisher Scientific, San Jose, CA, USA) was utilized alongside the airflow-assisted desorption electrospray ionization mass spectrometry imaging (AFADESI-MSI) platform, following a scanning protocol detailed in our previous study [44].

### 2.7. 16S rRNA Analysis of the Gut Microbiota

Fecal samples were used to extract total DNA with a commercial kit from Fangzhou Biosafety Technology (Guangzhou, China). The bacterial 16S rRNA gene is variable; we sequenced a sample of its V3–V4 region which is about 468 bp. The primers (338F and 806R) for the V3–V4 region selected for PCR amplification were 338F (ACTCCTACGGGAGGCAGCA) and 806R (GGACTACHVGGGTWTCTAAT) [45]. The PCR reaction was performed in a 50 μL mixture containing 25 μL of 2× Premix Taq (Takara, Dalian, China), 1 μL of each primer (10 μM), 50 ng of template DNA, and nuclease-free water to volume [46]. The thermocycling conditions were: 94 °C for 5 min, 30 cycles of 94 °C for 30 s, 52 °C for 30 s, 72 °C for 30 s, and a final extension at 72 °C for 10 min. We carried out 1.5% agarose gel electrophoresis at 120 V for 30 min, using a DL2000 DNA marker (Takara, Dalian, China) to check the fragment length and concentration of the PCR product. Concentrations of the PCR products were compared and subsequently mixed, following the data requirements, utilizing GeneTools Analysis Software version 4.03.05.0 (SynGene, Cambridge, UK). The library was prepared using the standard protocol associated with the ALFA-SEQ DNA library prep kit, and the sequencing of the amplicon library was conducted on the Illumina platform utilizing PE250 technology.

### 2.8. No-Targeted Metabolomics of Serum, Brain Tissue, and Feces

#### 2.8.1. Sample Pretreatment

A quantity of 50 mg of fecal matter was combined with 500 μL of chilled water, acetonitrile, and methanol in a ratio of 1:2:2 (*v*/*v*/*v*). This mixture underwent low-temperature homogenization for 5 min, followed by vigorous vortexing for 1 min and sonication at 40 kHz in an ice bath for 10 min. To denature the proteins, acetonitrile and methanol in a 1:1 (*v*/*v*) ratio were combined with serum in proportions of 300 μL solvent to 100 μL serum. Following this preparation, the mixture was subjected to vortexing for one minute, then sonicated at 40 kHz in an ice bath for ten minutes and subsequently kept at −20 °C for thirty minutes to enhance protein precipitation. The resulting supernatant was subsequently employed for metabolite analysis. For brain tissue, a chilled mixture of 300 μL containing methanol, acetonitrile, and water in same volumes (1:1:1, *v*/*v*/*v*) was prepared. This solution was homogenized at low temperatures for 10 min, followed by 1 min of vortex mixing, then subjected to sonication at 40 kHz in an ice bath for another 10 min and kept at −20 °C overnight. Each specimen underwent centrifugation at a speed of 15,000 rpm for a duration of 15 min at a temperature of 4 °C. The supernatant was meticulously drawn with a syringe, subsequently filtered using a 0.22-μm organic membrane, placed into a sample vial, and then examined via liquid chromatography–mass spectrometry. The sample pretreatment procedures were performed following the protocol with minor modifications [25].

#### 2.8.2. Sample Detection and Analysis

The metabolomics analysis was conducted using UHPLC-ESI-Q-Exactive Plus Orbitrap-MS (Thermo Fisher Scientific, San Jose, CA, USA) for the specific instrument conditions we referred to earlier research conducted by our group [47]. The mass spectrometry data were preprocessed using Compound Discoverer 3.3. Principal component analysis (PCA) was performed with the software SIMCA-P 14.1 (Umetrics AB, Umeå, Sweden). We identified primary and secondary mass spectrometry fragments of endogenous metabolites utilizing the HMDB (https://hmdb.ca/) databases, alongside our in-house metabolite fragmentation spectral libraries for the verification of MS2 spectra. The impact of GLRRTF on metabolic pathways was evaluated using MetaboAnalyst 6.0.

### 2.9. Network Pharmacology

The chemical structures of tryptophan-related metabolites were obtained from the PubChem database. The SwissTargetPrediction (https://www.swisstargetprediction.ch/, accessed on 10 October 2025) program determined that the compound structures tried were potential targets of tryptophan-related metabolites. To screen depression-related targets, “depression” was utilized as the keyword to search GeneCards (https://www.genecards.org/, accessed on 10 October 2025), OMIM (Online Mendelian Inheritance in Man) (https://omim.org/, accessed on 10 October 2025), and DisGeNET (https://disgenet.com/, accessed on 10 October 2025) datasets with the specification of organism as “Homo sapiens.” After removing duplicate entries, a non-redundant disease target library was constructed. The overlap of metabolite and disease targets showed that depression and metabolites have shared targets. Using Cytoscape version 3.10.3, a “metabolite–target” network diagram was created using Cytoscape with nodes representing metabolites and targets that interact. They were annotated using DAVID platform. KEGG enrichment analysis was performed to visualize key targets.

### 2.10. Western Blot Assay

Brain tissues were lysed for two hours on ice using NCM RIPA Buffer (NCM Biotech, Suzhou, China) supplemented with 1 mM PMSF and 1× phosphatase inhibitor cocktail (prepared from a 100× stock; both from Proteintech Group, Inc. (Wuhan, Hubei, China)). Following lysis, centrifugation was performed on the lysate at 12,000 g for 10 min at 4 °C to yield the supernatant. Proteins (10 μg/well) were separated on 10% SDS-PAGE with a prestained ladder (Thermo Fisher Scientific, Waltham, MA, USA, Cat# 26616) in 1× Tris-Glycine-0.1% SDS buffer at 80 V (stacking gel) and then 120 V (resolving gel), followed by transfer to PVDF membranes. The membranes were blocked with a 5% acid whey protein solution for one hour, followed by overnight incubation with primary antibodies at 4 °C. The primary antibodies used were TPH2 (1:2000), DDC (1:800), BDNF (1:800), TrkB (1:2000), p-PI3K (1:1000), PI3K (1:2000), p-AKT (1:2000), AKT (1:5000), and GAPDH (1:10,000). A one-hour incubation with secondary antibodies followed. Protein bands were visualized using the Tanon 5200 ECL detection system (Tanon, Science & Technology Co., Ltd., Shanghai, China), and semi-quantitative analysis was performed with Fiji (version 20250529-2217, ImageJ distribution). The expression levels of TPH2, DDC, BDNF, and TrkB were determined by normalizing the respective protein expression levels to that of GAPDH. For the assessment of relative expression levels of p-PI3K and PI3K, each was initially normalized to GAPDH, then p-PI3K/GAPDH was divided by PI3K/GAPDH for normalization. The procedure for treating p-AKT mirrored that of p-PI3K. The entire Western blot procedure was performed according to the standard protocol [48].

### 2.11. Data Processing and Analysis

For the analysis, SPSS version 31.0.0.0 and GraphPad Prism version 10.5 were utilized. Data are expressed as mean ± standard deviation (X ± SD). The Shapiro–Wilk H test was specifically employed to evaluate the alpha diversity of gut microbiota. A one-way ANOVA was performed for group comparisons on other data, followed by the LSD-*t* test for intergroup differences. A *p* value below 0.05 was deemed statistically significant.

## 3. Results

### 3.1. Preliminary Identification of the Chemical Components of GLRRTF

A total of 72 chemical components in the *G. lucidum* and *R. roxburghii* extracts formulation were identified, including six amino acids, nine organic acids, nine fatty acids and their derivatives, eight flavonoids, six saccharides, six alkaloids, sixteen terpenoids, five phenols, one anthraquinone, two aromatic aldehydes, three aromatic ketones, and one vitamin (Table S1). It is worth noting that tryptophan was identified.

### 3.2. Effects of GLRRTF on CUMS-Induced Depressive-like Behaviors in Mice

**Figure 1:**
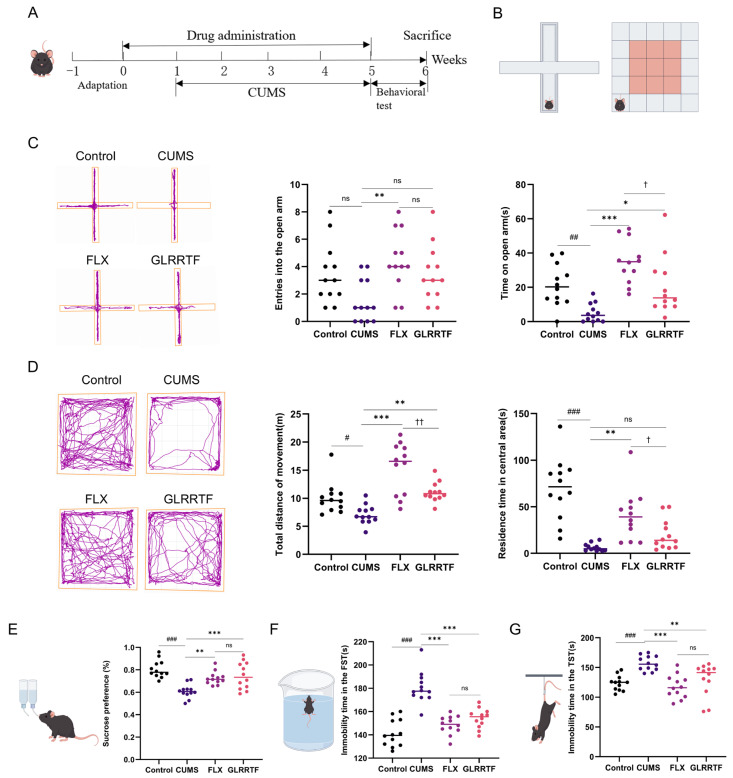
GLRRTF enhances behaviors associated with depression in mice subjected to CUMS. (**A**) Design of the CUMS experiment; (**B**) schematic representation of the EPMT and OFT; (**C**) trajectory chart showing entries into the open arm and duration spent in the open arm in the EPMT; (**D**) trajectory chart detailing total movement distance and time spent in the central zone in the OFT; (**E**) analysis of GLRRTF’s influence in the SPT; (**F**) recorded immobility duration in the FST; (**G**) immobility duration observed in the TST (*n* = 12). Prior training before the final test day was performed only for SPT (48 h sucrose habituation) and FST (15 min pretest swim 24 h before); no prior training was given for OFT, EPMT, or TST. All schematic diagrams are from Figdraw. ^#^ *p* < 0.05, ^##^ *p* < 0.01, ^###^ *p* < 0.001, CUMS vs. control group; * *p* < 0.05, ** *p* < 0.01, *** *p* < 0.001, GLRRTF vs. CUMS group; ^†^ *p* < 0.05, ^††^ *p* < 0.01, GLRRTF vs. FLX group. ns, no significant difference.

Following the CUMS modeling, depressive-like behaviors were observed in the mice. In the EPMT (Figure 1C), the CUMS group made fewer entries into the open arms compared to the control group. The drug-treated groups exhibited more entries than the CUMS group, but only the increase in the FLX group was statistically significant. The CUMS group showed a significant reduction in time spent in the open arms compared to the control group. In contrast, the FLX and GLRRTF groups showed an increase compared to the CUMS group. The OFT was employed to evaluate mice locomotion and exploratory behavior. The CUMS group exhibited reduced total movement compared to the control group (Figure 1D), while the FLX and GLRRTF groups showed increased movement. The CUMS group spent less time in the central area compared to the control group, while the FLX group showed significant improvement. In contrast, the GLRRTF group exhibited a less marked enhancement. In the SPT, mice in the CUMS group showed significantly reduced sucrose intake compared to the control group (Figure 1E). The FLX and GLRRTF groups showed a higher sucrose preference than the CUMS group. To further explore the impact of each drug treatment on mice experiencing stress, we performed the TST and the FST. The CUMS group exhibited a longer immobility duration compared to the control group, whereas both the FLX and GLRRTF groups showed reduced immobility relative to the CUMS group.

### 3.3. Effects of GLRRTF Intervention on Histopathology of CUMS-Induced Depressive Mice

**Figure 2:**
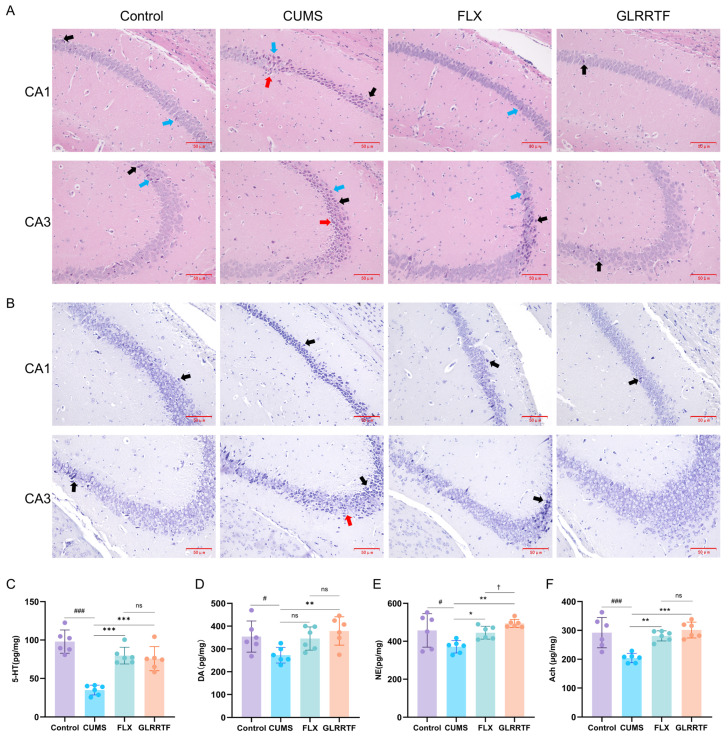
Impact of GLRRTF on hippocampal neuronal injury and levels of depression-associated markers in mice subjected to CUMS. (**A**) H & E staining (n = 3); arrows indicate: black arrow = apoptosis (shrunken, dark cytoplasm, pyknotic nucleus); blue arrow = pyknosis (small dark nucleus, normal cytoplasm); red arrow = necrosis (red cytoplasm, karyolysis, edema). (**B**) Nissl staining (n = 3); arrows indicate: black arrow = apoptosis (cell shrinkage, pyknosis, reduced Nissl); red arrow = necrosis (absent Nissl, pale cytoplasm, indistinct borders). (**C**) 5-HT levels (n = 6). (**D**) DA levels (n = 6). (**E**) NE content (n = 6). (**F**) Ach levels (n = 6). Scale bar = 50 μm. ^#^ *p* < 0.05, ^###^ *p* < 0.001, CUMS vs. control group; * *p* < 0.05, ** *p* < 0.01, *** *p* < 0.001, GLRRTF vs. CUMS group; ^†^ *p* < 0.05, GLRRTF vs. FLX group. ns, no significant difference.

Under H & E staining (Figure 2A), the hippocampal structure in the control group was normal; neurons were abundant, closely and orderly arranged, with clear contours and regular shapes, and occasional apoptotic or pyknotic neurons were observed. In the CUMS group, the hippocampal structure was severely disrupted, neuronal number decreased, and many neurons showed apoptosis (shrunken cell body, pyknotic nucleus, deep eosinophilic cytoplasm) and pyknosis (small dark nucleus, relatively normal cytoplasm); necrotic cells (homogeneous red cytoplasm, karyolysis, surrounding edema, indistinct cell borders) were also present, and the remaining neurons were loosely and disorderly arranged. In the FLX group, the hippocampal structure was largely normal, neurons were orderly arranged, a small number of neurons showed apoptosis or pyknosis, and necrosis was not observed. In the GLRRTF group, hippocampal injury was ameliorated, neurons were orderly arranged, occasional apoptotic or pyknotic neurons were seen, and neurons were not detected.

After Nissl staining (Figure 2B), hippocampal neurons in the control group were abundant, with Nissl bodies deeply stained, numerous, and densely distributed in the cytoplasm; occasional apoptotic neurons were seen, but no pyknosis was identified. In the CUMS group, hippocampal neurons were sparsely arranged and reduced in number, cytoplasmic staining was pale, and many neurons showed apoptosis (cell shrinkage, pyknosis, reduced Nissl substance) and necrosis (marked loss or absence of Nissl substance, pale cytoplasm, indistinct cell borders); no pyknosis was identified. Nissl bodies were few. In the FLX group, many neurons were densely distributed, with deep basophilic cytoplasmic staining and numerous, prominent Nissl bodies; however, a small number of neurons still showed apoptosis, and necrosis was not observed; no pyknosis was identified. In the GLRRTF group, hippocampal neurons were numerous and plump, Nissl bodies were abundant and densely distributed with deep basophilic staining, cells were relatively closely arranged, occasional apoptotic neurons were seen, and necrosis was absent; no pyknosis was identified.

### 3.4. Effects of GLRRTF on Neurotransmitters in CUMS-Induced Depressive Mice

Alterations in neurotransmitters frequently accompany depression. As illustrated in Figure 2C–F, the concentrations of 5-HT, DA, NE, and Ach in the brain of mice subjected to CUMS were lower than those in the control group. In contrast, the oral administration of GLRRTF for 35 consecutive days resulted in a notable enhancement of neurotransmitter concentrations, and the efficacy of GLRRTF surpassed that of FLX. These results indicate that GLRRTF might help reduce symptoms of anxiety and depression by regulating the levels of neurotransmitters.

### 3.5. AFADESI-MSI Analysis

**Figure 3:**
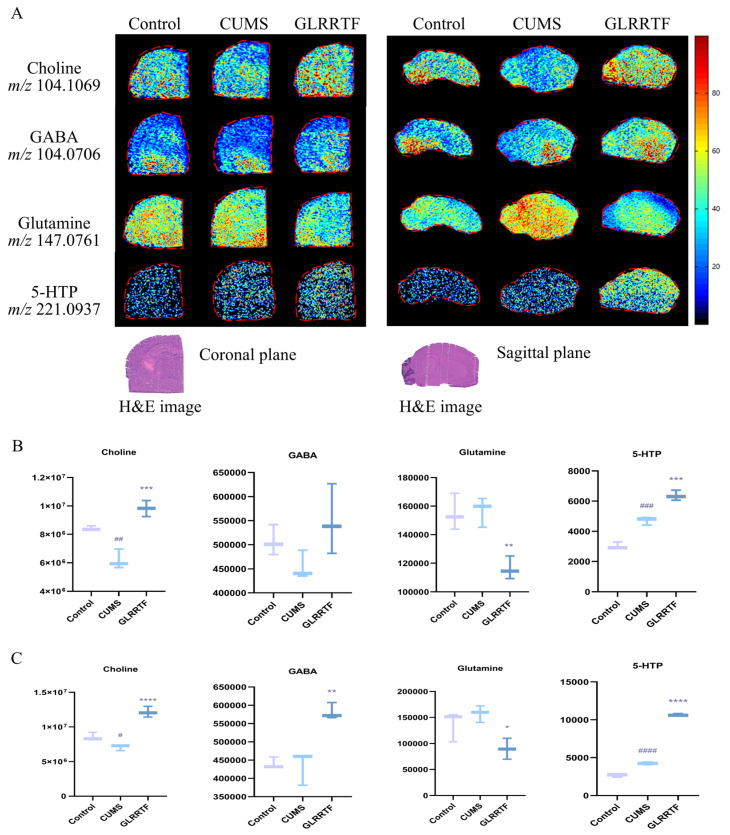
Analysis of targeted imaging of crucial differential metabolites, utilizing liquid chromatography tandem mass spectrometry screening alongside mass spectrometry imaging. (**A**) Visual representations of neurotransmitters and metabolites exhibiting variations. (**B**) The content differences in choline, GABA, glutamine, 5-HTP in coronal plane of brain section (n = 3). (**C**) The content differences in choline, GABA, glutamine, 5-HTP in sagittal plane of brain section (n = 3). ^#^ *p* < 0.05, ^##^ *p* < 0.01, ^###^ *p* < 0.001, ^####^ *p* < 0.0001, CUMS vs. control group; * *p* < 0.05, ** *p* < 0.01, *** *p* < 0.001, **** *p* < 0.0001, GLRRTF vs. CUMS group.

Four typical neurotransmitters or related metabolites were identified through mass spectrometry imaging analysis (Figure 3A). The imaging results revealed that the region with the highest density of GABA distribution was the basal ganglia, which is consistent with theoretical expectations. Other neurotransmitters and metabolites did not exhibit distinct regional distributions and were generally distributed throughout the entire brain. The levels of choline and GABA decreased in the CUMS group (Figure 3B,C) while GLRRTF administration increased their levels. Choline showed changes, with decreased levels in the CUMS group and increased levels in the GLRRTF group in both the coronal and sagittal planes. Regarding GABA, the CUMS group’s levels were reduced in the coronal plane compared to the control group but showed an increase in the sagittal plane. Following the administration of GLRRTF, GABA levels rose; however, differences between the CUMS and GLRRTF groups were only recorded in the sagittal plane sections. Although glutamine is not a typical neurotransmitter, it is a direct precursor of two important neurotransmitters in the brain, glutamate and GABA. Its level decreased after GLRRTF administration. Considering the increase in GABA level and the lack of differences in glutamate levels among the three groups in non-targeted metabolomics, it can be inferred that GLRRTF administration promotes the production of GABA. 5-HTP is a precursor of 5-HT, an important neurotransmitter affecting depression. Mass spectrometry imaging revealed that 5-HTP levels were greater in the CUMS group compared to the control group, and they rose further following the administration of GLRRTF.

### 3.6. Effects of GLRRTF on Gut Microbiota Dysbiosis in Depressive Mice

**Figure 4:**
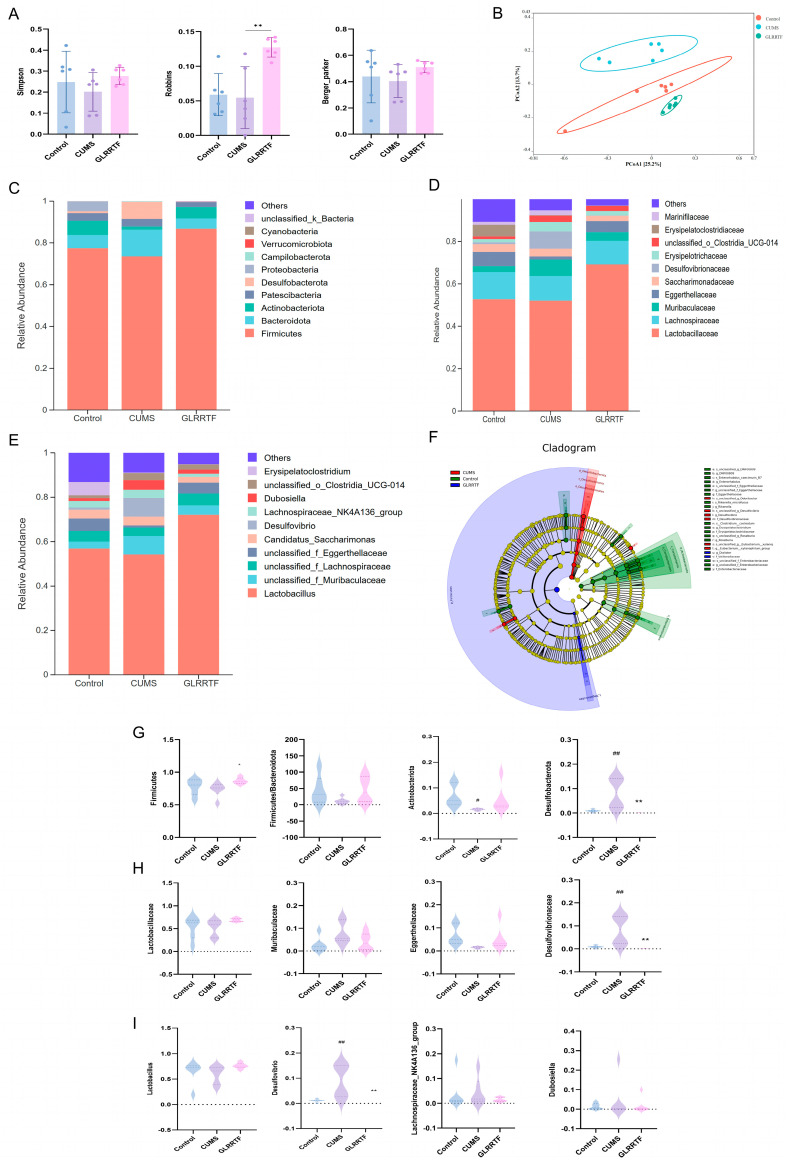
Influence of GLRRTF on the gut microbiota imbalance in mice exhibiting depression-like behavior induced by CUMS. (**A**) Comparison of α-diversity indices (Simpson, Robbins, Berger–Parker) in the gut microbiota (n = 6). (**B**) PCoA analysis based on OTU exhibited differences in β-diversity; each point denotes a sample. (**C**–**E**) Average relative abundance of microbial communities at the phylum, family, and genus levels. (**F**) Cladogram illustrates the comparison of bacterial taxa in the gut microbiota using LEfSe among the three groups at the OUT level (LDA > 3.0 and *p* < 0.05). (**G**–**I**) Average relative abundance of microbiota at the phylum, family, and genus levels (n = 6). ^#^ *p* < 0.05, ^##^ *p* < 0.01, CUMS vs. control group; * *p* < 0.05, ** *p* < 0.01, GLRRTF vs. CUMS group.

Ecological dysbiosis of the gut is a notable feature associated with depression. We assessed the influence of GLRRTF on gut microbiota dysbiosis in mice subjected to CUMS. The analysis of alpha diversity provides insights into the richness of the gut microbiota within a particular group, using the Simpson, Robbins, and Berger–Parker indices for evaluation (Figure 4A). While GLRRTF appeared to mitigate the decline in alpha diversity induced by CUMS, no statistical differences were observed in the changes in the Simpson and Berger–Parker indices across groups. In contrast, the Robbins index for the GLRRTF group exhibited an increase compared to the CUMS group. Beta diversity assessment reflects disparities in the structural diversity of gut microbiota among samples. Utilizing the PCoA model for analysis indicated that the three groups were distinctly separated, with the GLRRTF group showing a closer resemblance to the control group (Figure 4B). These findings suggest that the GLRRTF intervention effectively restored diversity in the gut microbiota within CUMS-induced mice. Further analysis of the gut microbiota’s taxonomic composition highlighted the top 10 bacterial species in terms of relative abundance across three different taxonomic levels: phylum, family, and genus (Figure 4C–E). At the phylum level, Desulfobacterota, Patescibacteria, Actinobacteriota, Bacteroidota, and Firmicutes emerged as the predominant phyla in all groups, contributing to over 90% of the total bacterial community (Figure 4C). Notably, the relative abundances of Firmicutes and Actinobacteriota were reduced in the CUMS group (Figure 4G), with the reduction in Actinobacteriota reaching statistical significance. GLRRTF intervention reversed the reductions in the relative abundances of these two phyla, and the effect on Firmicutes was statistically significant. The relative abundances of Bacteroidota and Desulfobacterota increased in the CUMS group, with the increase in Desulfobacterota being statistically significant. GLRRTF intervention reduced the relative abundances of these two phyla, and the reduction in Desulfobacterota was statistically significant. Additionally, the Firmicutes/Bacteroidota ratio decreased after CUMS modeling and increased in the GLRRTF group.

At the family level, the relative abundances of Lactobacillaceae and Eggerthellaceae in the CUMS group decreased (Figure 4H). GLRRTF intervention reversed these decreases, but neither change was statistically significant. Additionally, the relative abundances of Muribaculaceae and Desulfovibrionaceae increased in the CUMS group. GLRRTF intervention reduced the relative abundances of these bacteria, with the reduction in Desulfovibrionaceae being statistically significant. In the CUMS group, the relative abundance of *Lactobacillus* was reduced (Figure 4I). GLRRTF intervention reversed this reduction, but the change was not statistically significant. Moreover, the relative abundances of *Desulfovibrio*, *Lachnospiraceae_NK4A136_group*, *Dubosiella*, and unclassified_*o_Clostridia_UCG-014* increased in the CUMS group, and GLRRTF intervention could reduce the relative abundances of these bacteria. Among these genera, the harmful *Desulfovibrio* [49] showed a statistically significant increase in the CUMS group and a statistically significant decrease following GLRRTF intervention.

To distinguish the groups with effects from phylum to genus, LEfSe (linear discriminant analysis effect size) analysis was conducted (Figure 4F), leading to the creation of a cladogram (LDA score > 3.0). LEfSe analysis revealed significant differences in gut microbiota among the three groups from phylum to genus level. In the CUMS group, enriched taxa were mainly from Desulfobacterota (class Desulfovibrionia to genus Desulfovibrio). The control group enriched Actinobacteriota (family Eggerthellaceae) and Enterobacterales (family Enterobacteriaceae). GLRRTF intervention enriched Firmicutes (class Negativicutes to genus Dialister). These findings suggest that CUMS induced a marked shift in gut microbiota, which was reversed by GLRRTF. 

### 3.7. Effects of GLRRTF on Fecal Metabolites in Depressed Mice

**Figure 5:**
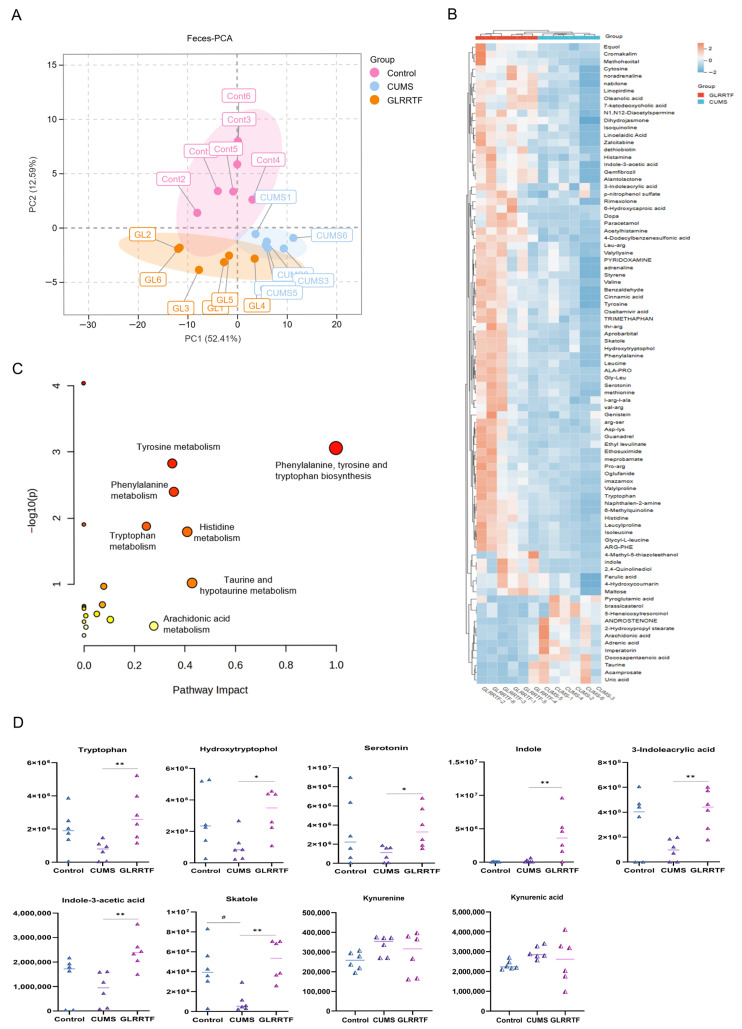
GLRRTF modulated the feces metabolome. (**A**) PCA diagram of feces; (**B**) heatmap of 87 differentially expressed metabolites in feces; (**C**) enrichment analysis of differentially expressed metabolites in feces; (**D**) change in the expression level of the 7 tryptophan-related metabolites with a significant difference (n = 6). ^#^ *p* < 0.05, CUMS vs. control group; * *p* < 0.05, ** *p* < 0.01, GLRRTF vs. CUMS group.

To gain further insights into how GLRRTF influences fecal metabolism, we utilized an LC-MS platform for the examination of fecal metabolites. The PCA-X model was applied to investigate the metabolite profiles from three sample groups: control, CUMS, and GLRRTF. As illustrated in Figure 5A, the three groups exhibited considerable separation. Based on the criteria of variable importance in projection > 1, *p* < 0.05, and Fc > 2 or Fc < 0.5, we identified 87 metabolites that differed in the feces. A heatmap was generated to depict the variations in these differential metabolites in the CUMS and GLRRTF groups (Figure 5B). Among the identified metabolites, 12 were found to be upregulated, while 75 were downregulated. Additionally, an analysis of the enrichment of metabolic pathways related to the differential metabolites revealed that 20 metabolic pathways were influenced in the fecal matter. Particularly, pathways related to the biosynthesis of phenylalanine, tyrosine, and tryptophan, as well as tyrosine metabolism, phenylalanine metabolism, tryptophan metabolism, histidine metabolism, taurine and hypotaurine metabolism, and arachidonic acid metabolism were notably impacted (Figure 5C). As shown in Figure 5D, a separate analysis of seven metabolites in the tryptophan metabolic pathway was conducted. In the CUMS group, the levels of six metabolites were lower than those in the control group, but only the decrease in skatole was statistically significant.However, after GLRRTF intervention, the levels of all seven metabolites increased. Although no differential metabolites were screened in the kynurenine pathway, to analyze the overall changes in tryptophan metabolism, the changes in kynurenine and kynurenic acid were also analyzed. The levels of both kynurenine and kynurenic acid increased after CUMS modeling and decreased after GLRRTF intervention.

### 3.8. Serum Metabolomics Analysis

**Figure 6:**
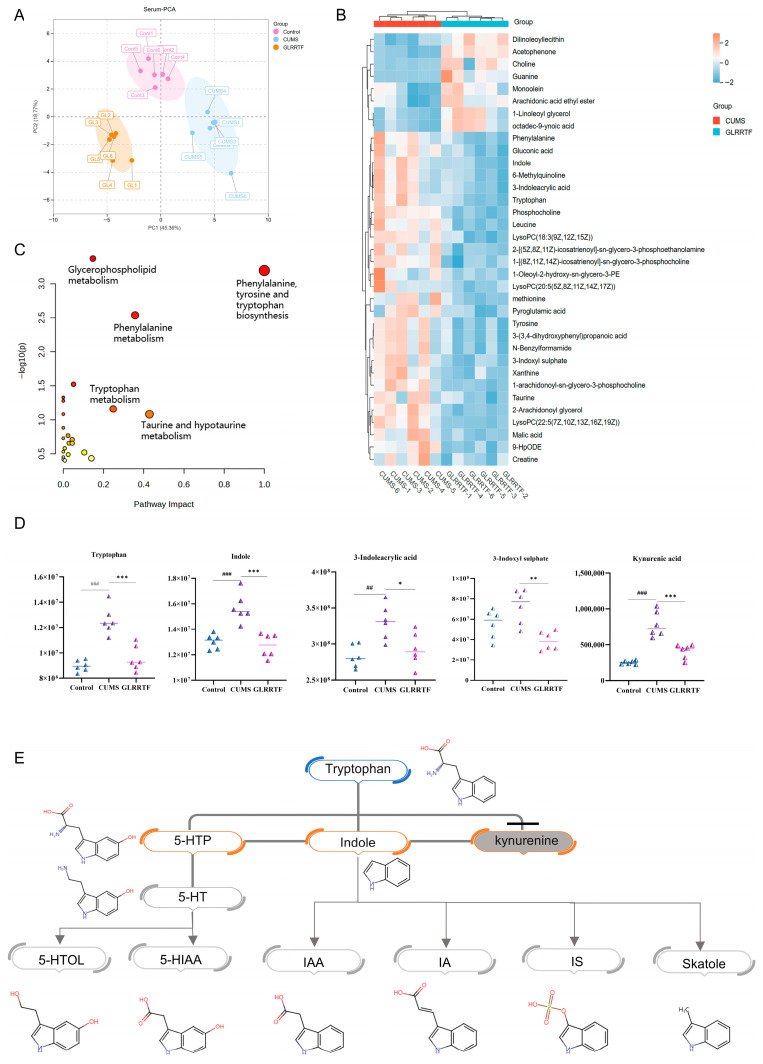
The serum metabolome was modulated by GLRRTF, with representative metabolic changes illustrated in the metabolomics analysis. (**A**) PCA representation of serum; (**B**) heatmap displaying 35 metabolites that showed differential expression in serum; (**C**) enrichment analysis for the differentially expressed metabolites in serum; (**D**) variation in expression levels of four tryptophan-related metabolites with notable differences (n = 6); (**E**) chart illustrating the variations in tryptophan metabolites found in feces, serum, and brain. ^##^ *p* < 0.01, ^###^ *p* < 0.001, CUMS vs. control group; * *p* < 0.05, ** *p* < 0.01, *** *p* < 0.001, GLRRTF vs. CUMS group. 5-HTP, 5-hydroxytryptophan; 5-HT, serotonin; 5-HIAA, 5-hydroxyindole-3-acetic acid; 5-HTOL, hydroxytryptophol; IAA, indole-3-acetic acid; IA, 3-indoleacrylic acid; IS, 3-indoxyl sulphate.

PCA was used to analyze the metabolic profile data, highlighting differences in serum samples obtained from diverse groups of mice (Figure 6A). Different from the fecal metabolomics conditions, Fc values were greater than 1.2 or less than 0.8. A total of 35 metabolites showed significant differences in serum levels. To depict the expression levels of these metabolites among different sample groups, a heatmap was generated. Of the metabolites identified, eight were observed to be upregulated, while 27 demonstrated downregulation (Figure 6B). These findings suggested that the GLRRTF intervention mitigated metabolic disturbances in mice subjected to the CUMS model. The analysis of metabolic pathway enrichment revealed a total of 23 metabolic pathways present in the serum. Importantly, pathways such as the biosynthesis of phenylalanine, tyrosine, and tryptophan, glycerophospholipid metabolism, and phenylalanine were notably impacted in the serum (Figure 6C). The tryptophan metabolism pathway did not reach statistical significance (*p* > 0.05). Tryptophan metabolomic analysis (Figure 6D) showed that, compared with the control group, the CUMS group had higher levels of tryptophan, indole, and 3-indoleacrylic acid (*p* < 0.01 or *p* < 0.001). 3-Indoxyl sulfate levels also increased but did not reach statistical significance. After GLRRTF treatment, the levels of all four metabolites dropped significantly (*p* < 0.05, *p* < 0.01, or *p* < 0.001) and returned to levels comparable to those in the control group, except for 3-indoxyl sulfate, which fell even below control levels. Kynurenic acid was not among the screened differential metabolites, but it was also analyzed to comprehensively evaluate the changes in tryptophan metabolism. The results showed that its level increased after CUMS modeling and decreased after GLRRTF intervention.

### 3.9. Analysis of Brain Tissue Metabolomics

**Figure 7:**
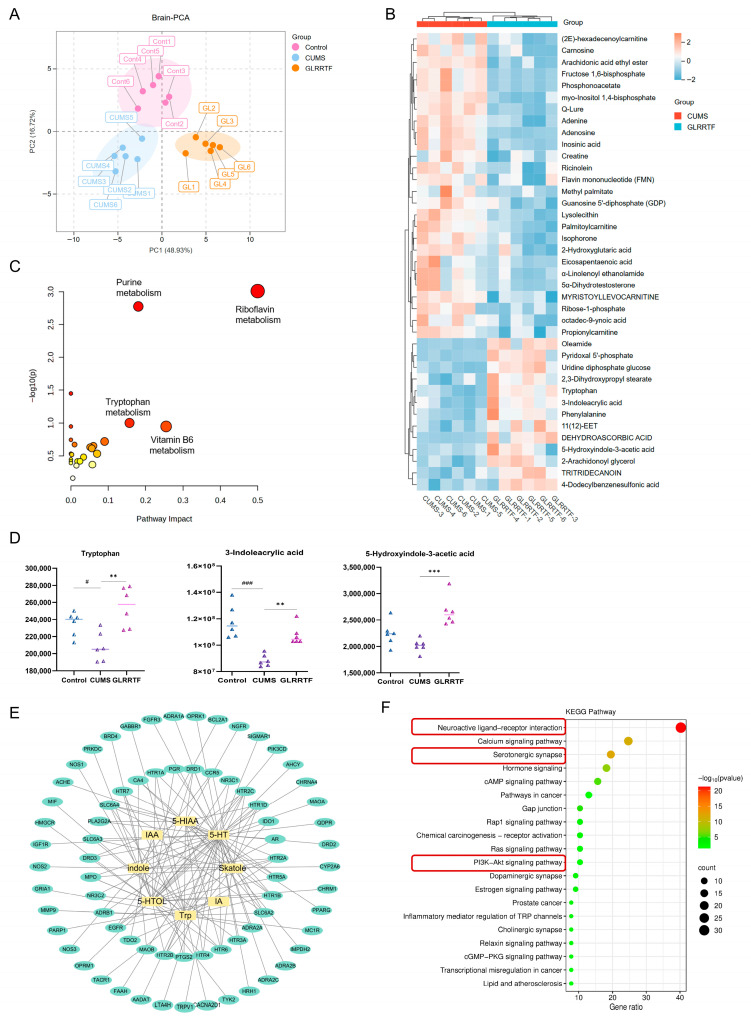
The modulation of the brain metabolome by GLRRTF, along with a network pharmacological analysis concerning its effects on depression treatment, is examined. (**A**) The PCA diagram representing brain data; (**B**) a heatmap illustrating 39 metabolites with differential expression in the brain; (**C**) an enrichment analysis focused on the differentially expressed metabolites in the brain; (**D**) variations in the expression levels of three tryptophan-related metabolites showing significant differences (n = 6); (**E**) the anti-depression activity network featuring tryptophan-related metabolites linked to GLRRTF; (**F**) KEGG enrichment analysis of the potential targets associated with tryptophan-related metabolites from GLRRTF in the context of depression. ^#^ *p* < 0.05, ^###^ *p* < 0.001, CUMS vs. control group; ** *p* < 0.01, *** *p* < 0.001, GLRRTF vs. CUMS group.

To confirm the model’s reliability, PCA was implemented to assess the metabolic profile data without relying on group classifications. The findings indicated a distinction in the metabolic profiles of brain tissue across various groups of mice (Figure 7A). Utilizing the same criteria as serum metabolomics, a total of 39 differential metabolites were detected. To represent the different expression levels of metabolites across the sample groups, a heatmap was generated. Out of these metabolites, 13 were found to be upregulated while 26 were downregulated (Figure 7B). These outcomes suggested that the GLRRTF intervention successfully mitigated metabolic disorders in mice subjected to the CUMS model. An analysis of metabolic pathway enrichment indicated 24 metabolic pathways impacted in the brain tissue, including riboflavin metabolism and purine metabolism. Notably the tryptophan metabolism pathway did not reach statistical significance (*p* > 0.05) (Figure 7C). Additionally, an individual analysis of three metabolites involved in tryptophan metabolism (Figure 7D) demonstrated that the levels of metabolites in the CUMS group were reduced in comparison to the control group; however, following the GLRRTF intervention, these levels rose.

### 3.10. Network Pharmacology

To investigate how GLRRTF functions to prevent depression, we performed a network pharmacology analysis. For the nine tryptophan-related metabolites screened from fecal, serum, and brain metabolomics, 252 related targets were collected from SwissTargetPrediction. Subsequently, 1918 targets associated with depression were collected. After intersection analysis of these data, 77 potential targets of GLRRTF for preventing depression were identified. Figure 7E,F show eight tryptophan-related metabolites (the predicted targets probabilities of 3-indoxyl sulphate (IS) were all zero, so it was not adopted), 77 potential targets, and 20 enriched KEGG pathways. The KEGG enrichment analysis revealed that 20 pathways were notably influenced, among which the neuroactive ligand–receptor interaction, serotonergic synapse, PI3K/AKT signaling pathway, dopaminergic synapse, and cholinergic synapse were closely related to depression. Next, the mechanism of action of GLRRTF will be further verified based on the neuroactive ligand–receptor interaction, serotonergic synapse, and the PI3K/AKT signaling pathway.

### 3.11. Western Blot

**Figure 8:**
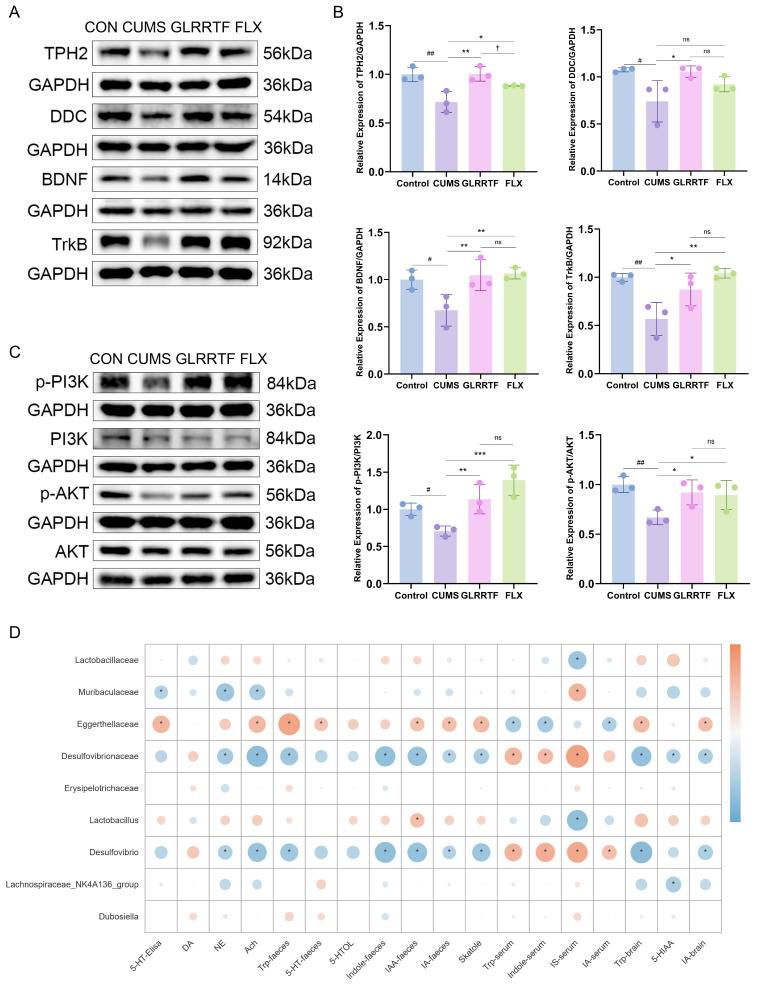
Results from Western blot analysis of proteins closely linked to 5-HT synthesis and the BDNF system, along with correlation analysis among neurotransmitter levels, proteins, metabolites, and gut microbiota. (**A**,**C**) The expression levels of TPH2, DDC, BDNF, TrkB, p-PI3K, and p-AKT were evaluated using WB. (**B**) The scatter bar chart illustrates the statistical findings of various proteins (n = 3). (**D**) Correlation analysis was performed on neurotransmitter levels, metabolites, and gut microbiota at family and genus levels, using the Spearman test in the Metware Cloud. ^#^ *p* < 0.05, ^##^ *p* < 0.01, CUMS vs. control group; * *p* < 0.05, ** *p* < 0.01, *** *p* < 0.001, GLRRTF vs. CUMS group; ^†^ *p* < 0.05, GLRRTF vs. FLX group. ns, no significant difference.

Western blot analysis (Figure 8A–C) demonstrated downregulation of protein expression levels for TPH2, DDC, BDNF, TrkB, p-PI3K, and p-AKT in the brain tissues of mice subjected to CUMS-induced depression. However, following treatment with GLRRTF and FLX, these effects were alleviated. It is important to note that the recovery of TPH2 and DDC levels after FLX treatment was less pronounced compared to the GLRRTF group.

### 3.12. Comprehensive Analysis of Gut Microbiota and Metabolites

To gain deeper insight into the intricate mechanism through which GLRRTF mitigates behaviors resembling depression, we performed an extensive analysis of the relationships among neurotransmitters, tryptophan-related metabolites in brain tissue, tryptophan-related metabolites in serum, tryptophan-related metabolites in feces, and the microbiota at the family and genus levels that changed after GLRRTF administration (Figure 8D). The results showed that Eggerthellaceae had positive correlations with multiple neurotransmitters and tryptophan-related metabolites. Desulfovibrionaceae and *Desulfovibrio* had negative correlations with multiple neurotransmitters and tryptophan-related metabolites. However, the correlation of IS in serum was opposite to that of the remaining metabolites and neurotransmitters. In addition, Lactobacillaceae and *Lactobacillus* had negative correlations with IS. *Lactobacillus* had positive correlations with indole-3-acetic acid (IAA) in feces and tryptophan in serum. Muribaculaceae had negative correlations with 5-HT, NE, Ach, and 3-indoleacrylic acid (IA) in serum, and had a positive correlation with IS. The analysis of correlation between neurotransmitters/metabolites and gut microbiota revealed a strong association between gut microbiota and the development of depression.

## 4. Discussion

In this study, we demonstrated that GLRRTF prevents CUMS-induced depressive-like behaviors in mice. Mechanistically, GLRRTF restored neurotransmitter levels in the brain, modulated gut microbiota composition, and promoted tryptophan metabolism toward the 5-HT and indole pathways. Network pharmacology and Western blot validation further revealed that GLRRTF activated the BDNF/TrkB/PI3K/AKT signaling pathway.

We identified multiple compounds in GLRRTF by UHPLC-ESI-Q-Exactive Plus Orbitrap-MS. Many of them have been shown to produce antidepressant-like effects in animal models, including flavonoids (quercetin [50], kaempferol [51], apigenin [52], catechin [53], epicatechin [54]), phenolic acids (ellagic acid [55], ferulic acid [56]), and nine known Ganoderma triterpenoids (e.g., lucidenic acid B, ganodernoid D, ganosinensic acid A) [22]. Notably, tryptophan was also identified in GLRRTF. These compounds are known to act via monoaminergic modulation, anti-inflammation, or BDNF upregulation. Our own data, including increased tryptophan levels and activation of the BDNF pathway, are consistent with this picture, suggesting that these components may collectively contribute to the preventive effects of GLRRTF.

Mapping where brain metabolites are located is key to understanding depression [57]. Regular metabolomics cannot tell you where things are in the brain [58], so we used AFADESI-MSI to look at neurotransmitter distribution and to check the metabolites we found from untargeted metabolomics. The mouse brain is not symmetric—the left side is bigger [59] and more active in depression-related states like anhedonia and stress [60]. Also, the left amygdala handles emotional processing [61], and the left hippocampus controls long-term memory [62]. So, we cut the left hemisphere into coronal sections to cover regions like the hippocampus and used the right hemisphere for sagittal sections. Integrated analysis of AFADESI-MSI and ELISA showed that GLRRTF increased the conversion of glutamine to GABA and 5-HTP to 5-HT. Choline and acetylcholine levels also increased. GABA is known to play a role in depression [63], and glutamine is part of the glutamate/GABA–glutamine cycle; disruption of this cycle is linked to depression [64]. The cholinergic system also plays an important role in mood regulation [65]. 5-HTP is the direct precursor of serotonin (5-HT); its synthesis from tryptophan is the rate-limiting step in 5-HT biosynthesis [66]. Since 5-HTP and 5-HT went up in the GLRRTF group, we think that GLRRTF helps turn tryptophan into 5-HTP and then into 5-HT, which helps prevent depression. We then checked the enzymes involved in that pathway to confirm this. Thus, GLRRTF appears to regulate depression-related neurotransmitter pathways.

GBA plays a role in neurogenesis and depression by influencing the levels of tryptophan and BDNF [67]. We hypothesize that tryptophan metabolism and BDNF expression are important targets for GLRRTF to prevent depression induced by CUMS in mice. Next, we first investigated which gut microbiota have an effect on tryptophan metabolism in mice. The relative abundances of *Lactobacillus* and *Desulfovibrio* are associated with depressive-like behavioral disorders in both murine models and human studies [68,69]. In our data, the change in *Lactobacillus* did not reach statistical significance (*p* > 0.05), but we did see a trend toward reversal in the GLRRTF group compared to the CUMS group (mean relative abundance went up by 29%). This trend is directionally consistent with earlier work showing that the Radix Bupleuri–Radix Paeoniae Alba herb pair increases *Lactobacillus* [70]. In contrast, GLRRTF significantly reduced *Desulfovibrio* abundance (*p* < 0.05), which agrees with the report that a tryptophan-rich diet lowers *Desulfovibrio* while raising *Lactobacillus* [71]. The *Lactobacillus* genus has been proposed to influence depression via tryptophan metabolism [72]. That said, given the lack of statistical significance in our study, its role in the effect of preventing depression of GLRRTF is probably modest. In the context of this research, the enhancement of fecal tryptophan levels by GLRRTF may be connected to the reduced presence of specific bacteria in the gut that utilize tryptophan [73]. The GLRRTF intervention has been shown to reduce the relative abundances of Muribaculaceae, *Desulfovibrio, Lachnospiraceae_NK4A136_group*, and *Dubosiella*. The above results are in keeping with the findings of a study that comprehensively outlines the microbial changes in depression, characterizing them as pathological dysbiosis [68]. Spearman analysis shows that *Desulfovibrio* may be a candidate bacterium for decomposing gut tryptophan, and it is positively correlated with depression [74], but GLRRTF shows an inhibitory effect. Conversely, certain gut microorganisms have the potential to elevate the serum tryptophan levels [75]. Consuming GLRRTF may create a supportive environment in the gut for the proliferation of advantageous bacteria, which could enhance the production of tryptophan within the gastrointestinal tract. While we examined a number of bacteria that might influence depression in mice through their involvement in the regulation of gut tryptophan metabolism, additional in vitro studies and the transplantation of selected bacteria are essential for assessing their contributions to depression prevention in mice.

Next, we examined tryptophan metabolism in CUMS mice. Pathway enrichment analysis showed that GLRRTF enriched amino acid metabolism, particularly tryptophan. Tryptophan metabolism is linked to chronic stress [76], and CUMS reduces hippocampal tryptophan and 5-HT [77]. A tryptophan-rich diet inhibits the kynurenine pathway, shifting tryptophan toward the 5-HT and indole pathways, and increases BDNF expression [78] consistent with our findings. In our study, GLRRTF itself contains tryptophan, suggesting that it can serve as a direct source. Interestingly, tryptophan metabolism differed across the gut, serum, and brain compartments in CUMS mice: fecal and brain tryptophan decreased, while serum tryptophan increased. This dissociation suggests that despite abundant circulating tryptophan, its entry into the brain was restricted—possibly due to altered blood–brain barrier transport or impaired central utilization [79]. After GLRRTF treatment, serum tryptophan fell while brain tryptophan recovered, pointing to improved brain uptake. Additionally, serum indole derivatives and 3-indoxyl sulfate (a hepatic detoxification product) decreased, indicating that GLRRTF enhanced the host ‘s capacity to clear microbial metabolites [80]. Notably, no differential metabolites of the kynurenine pathway were detected in any metabolomics dataset. Therefore, we hypothesize that GLRRTF promotes tryptophan metabolism via the 5-HT and indole pathways rather than the kynurenine pathway [81].

To identify the key pathways through which GLRRTF regulates tryptophan metabolism, we performed network pharmacology analysis using nine tryptophan-related metabolites identified by metabolomics. KEGG enrichment revealed three pathways relevant to depression: neuroactive ligand–receptor interaction, serotonergic synapse, and PI3K/AKT signaling. We then experimentally validated each pathway using complementary methods. Neurotransmitter levels (5-HT, GABA, NE, ACh, DA) were measured by ELISA and mass spectrometry imaging to assess the neuroactive ligand–receptor pathway. For the serotonergic synapse pathway, we examined the two rate-limiting enzymes in serotonin synthesis: TPH2 and DDC. The BDNF/TrkB/PI3K/AKT cascade was analyzed by Western blot. Tryptophan is converted to 5-HTP by TPH2, and then to 5-HT by DDC. In the CUMS group, AFADESI-MSI showed an accumulation of 5-HTP, suggesting that DDC activity was suppressed under chronic stress, thereby blocking the conversion of 5-HTP to 5-HT. GLRRTF upregulated both TPH2 and DDC, indicating that it facilitates tryptophan-to-5-HT conversion and raises 5-HT levels. In contrast, FLX had a weaker effect on TPH2 and DDC, which is consistent with its mechanism as a selective 5-HT reuptake inhibitor—it does not directly affect serotonin synthesis. 5-HT is known to promote BDNF expression [82]. Reduced BDNF levels in the hippocampus and prefrontal cortex are associated with depression [83], and the BDNF/TrkB system is involved in depressive pathophysiology [84]. BDNF binding to TrkB leads to TrkB phosphorylation and activates downstream signaling, including the PI3K/AKT pathway, which is critical for synaptic plasticity [16]. In our study, GLRRTF significantly increased BDNF, TrkB, p-PI3K, and p-AKT, suggesting that it may promote neuronal repair and survival. Together, these results support that GLRRTF prevents depressive-like behaviors by enhancing serotonin synthesis and activating the BDNF/TrkB/PI3K/AKT pathway.

Both FLX and GLRRTF reversed CUMS-induced changes but differed in their profiles. FLX performed better in anxiety-related tests (EPMT, OFT), while GLRRTF reduced immobility more in the FST and TST; both similarly restored sucrose preference. GLRRTF elevated 5-HT, DA, NE, and ACh more than FLX and largely normalized hippocampal histology, whereas FLX left some degeneration. This may be explained by gut dysbiosis impairing FLX efficacy [85]. Western blot showed comparable BDNF/p-AKT between groups; FLX better recovered TrkB/p-PI3K, and GLRRTF upregulated TPH2/DDC more. These differences align with FLX’s mechanism as a 5-HT reuptake inhibitor and its known anti-anxiety properties [86]. The FLX group was not included in multi-omics analyses (brain imaging, 16S sequencing, metabolomics) due to budget constraints. Those assays focused on GLRRTF’s mechanism rather than direct comparison with FLX. Previous work has shown that FLX alters gut microbiota [87] and plasma metabolites [88] in CUMS models, but whether GLRRTF acts similarly or differently remains unknown. Future studies with proper funding should address this.

Another limitation of this study is the use of only male mice. Depression is more prevalent in females—epidemiological studies consistently report that women are roughly two to three times as likely as men to develop major depressive disorder [89]. In addition, both gut microbiota composition and tryptophan metabolism show sex-related differences [90,91]. The main reason we used only male mice in this exploratory study was to avoid the potential confounding effects of the estrous cycle on behavioral and metabolic parameters [92], which might otherwise obscure the interpretation of GLRRTF’s preventive effects. It should be noted that most animal models of depression were initially developed using male rodents and were only later applied to females [93]—a common practice in preclinical mechanistic studies. That said, we recognize that this choice limits the generalizability of our findings to female populations. Future studies should therefore evaluate the preventive effects of GLRRTF in female mice. Despite the limits, our findings provide new insights into GLRRTF’s preventive effect against depression.

## 5. Conclusions

In a mouse model of chronic stress, GLRRTF prevented depressive-like behaviors, gut microbiota dysbiosis, and metabolic disturbances. Mechanistic analysis revealed that GLRRTF acted through the gut–brain axis: it reshaped gut microbiota composition, increased tryptophan levels in the gut, serum, and brain, and enhanced brain 5-HT synthesis by upregulating TPH2 and DDC. The formulation also activated the BDNF/TrkB/PI3K/AKT signaling pathway. Together, these findings position GLRRTF as a functional food formulation with potential for supporting mental health.

## Data Availability

The original contributions presented in this study are included in the article/Supplementary Materials. Further inquiries can be directed to the corresponding authors.

## Supplementary Materials

The following supporting information can be downloaded at: https://www.mdpi.com/article/10.3390/foods15091535/s1; Table S1: Tentative identification of chemical composition in GLRRTF.

## Abbreviations

The following abbreviations are used in this manuscript:

5-HIAA	5-hydroxyindole-3-acetic acid
5-HT (serotonin)	5-hydroxytryptamine
5-HTOL	hydroxytryptophol
5-HTP	5-hydroxytryptophan
Ach	acetylcholine
AFADESI-MSI	airflow-assisted desorption electrospray ionization mass spectrometry imaging
AKT	protein kinase B
BDNF	brain-derived neurotrophic factor
CUMS	chronic unpredictable mild stress
DA	dopamine
DDC	DOPA decarboxylase
ELISA	enzyme-linked immunosorbent assay
EPMT	elevated plus maze test
FLX	fluoxetine
FST	forced swimming test
GABA	gamma-aminobutyric acid
GAPDH	glyceraldehyde-3-phosphate dehydrogenase
GBA	gut–brain axis
GLRRTF	*Ganoderma lucidum* and *Rosa roxburghii* Tratt formulation
H&E	hematoxylin and eosin
HPA	hypothalamic–pituitary–adrenal
IA	3-indoleacrylic acid
IAA	indole-3-acetic acid
IS	3-indoxyl sulphate
NE	norepinephrine
OFT	open field test
PCA	principal component analysis
PI3K	phosphatidylinositol 3-kinase
SPT	sucrose preference test
TPH2	tryptophan hydroxylase 2
TrkB	tropomyosin receptor kinase B
TST	tail suspension test
VIP	variable importance in projection
