# Supplementary material for: The Ganoderma lucidum and Rosa roxburghii Tratt Formulation Prevents Depressive-like Behaviors in Mice by Modulating Tryptophan Metabolism via the Gut–Brain Axis and Upregulating the BDNF/TrkB/PI3K/AKT Pathway
Source: Foods. 2026 Apr 28;15(9):1535. doi: 10.3390/foods15091535 (PMC13163458; doi:10.3390/foods15091535)
Supplement: Supplementary file 1 [file foods-15-01535-s001.zip › foods-4248178-supplementary.pdf]

Table S1. Tentative identification of chemical composition in GLRRTF.

NO	Identification Name	Formula	Observed MS1(*m/z*)	RT (min)	MS2(*m/z*)
1	DL-Arginine	C6H14N4O2	175.1191	0.734	175.11905、130.09767、116.07092、70.06575、60.05632
2	Choline	C5H13NO	104.1074	0.786	104.10733、60.08144
3	5-Aminovaleric acid	C5H11NO2	118.087	0.844	119.09004、118.08646、59.04980、55.0549
4	Proline	C5H9NO2	116.0709	0.861	116.07088、70.06571、68.05006
5	Adenosine	C10H13N5O4	268.104	0.903	268.10614、137.06525、136.06183、97.02871、85.02882、57.03417
6	Adenine	C5H5N5	136.0619	0.904	137.04582、136.06194、119.03596
7	Citric acid	C6H8O7	191.019	0.911	191.01897、173.00893、129.01816、111.00751、87.00742、85.02813
8	2-methylene-4-oxo-pentanedioic acid	C6H6O5	203.0188	0.94	157.01323、71.01242、69.03316
9	Nicotinamide	C6H6N2O	262.1287	1.235	123.05552、80.04999
10	L-Tyrosine	C9H11NO3	182.0813	1.263	165.05467、147.04408、136.07578、123.04427、119.04939、95.04960、91.05471

11	L-Isoleucine	C6H13NO2	132.1021	1.345	86.09694、69.07049
12	Hypoxanthine	C5H4N4O	137.046	1.382	137.04581、119.03545、110.03518、94.04041
13	L-Norleucine	C6H13NO2	132.1021	1.43	87.10001、86.09693、69.07035
14	Itaconic acid	C5H6O4	305.0517	1.442	129.01825、85.02799
15	α, α-Trehalose	C12H22O11	341.1093	1.975	341.10980、179.05487、119.63366、113.02303、101.02325、89.02303、71.01244
16	D- (-)-Quinic acid	C7H12O6	191.0554	2.162	191.05531、85.02815
17	D- (+)-Maltose	C12H22O11	341.1093	2.188	179.05576、119.03377、113.02307、101.02298、89.02302、71.01247
18	Sucrose	C12H22O11	341.1092	2.588	179.05510、119.03402、113.02317、101.02292、89.02303
19	trans-3-Indoleacrylic acid	C11H9NO2	188.0707	4.605	188.07065、146.06006、144.08092、118.06538
20	D- (+)-Tryptophan	C11H12N2O2	205.0976	4.613	188.07118、146.06046、144.08105、118.06554
21	Ellagic acid	C14H6O8	303.0134	4.978	303.0134、285.00278、275.01859、257.00793、247.02367、229.01315、201.01826
22	Epicatechin	C15H14O6	291.0863	5.532	165.06481、147.04425、139.03906、123.04430

23	Emodin	C15H10O5	271.0604	6.369	271.06049、225.05504、197.06007、173.0601
24	2,3,4,9-Tetrahydro-1H-β-carboline-3-carboxylic acid	C12H12N2O2	217.0972	6.479	217.09653、171.09183、144.08081、74.02419
25	4-Hydroxybenzaldehyde	C7H6O2	123.0443	6.581	123.04429、95.04958
26	3,4-Dihydroxybenzaldehyde	C7H6O3	139.039	6.582	139.03903、111.04439、93.03396
27	4-Coumaric acid	C9H8O3	165.0547	6.583	165.05473、147.04413、119.04942、91.05472
28	4-Methoxybenzaldehyde	C8H8O2	137.0598	6.848	137.05977、122.03646、94.04177
29	Apigenin	C15H10O5	269.0456	7.018	270.05096、269.04593
30	Isovanillic acid	C8H8O4	169.05	7.149	169.04951、151.03912、125.05992、111.04433、93.03406、65.03919
32	1,4-Dihydroxy-2-naphthoic acid	C11H8O4	203.0343	7.698	203.03459、159.04456、147.04427
33	Sinapinic acid	C11H12O5	207.0656	7.895	207.06569、192.04175、175.03941、147.04440、119.04957、91.05466
34	Catechin	C15H14O6	289.0718	8.121	289.07211、245.08203、125.02331、123.04401、109.02828

35	3,5-Dihydroxy-2-(4-hydroxyphenyl)-4-oxo-3,4-dihydro-2H-chromen-7-yl hexopyranoside	C21H22O11	449.1093	8.215	449.10971、287.05576、259.06143、125.02323
36	3',4'-Dimethoxyacetophenone	C10H12O3	181.086	8.284	181.086、137.05986、107.04955
37	2,3-Dihydro-1-benzofuran-2-carboxylic acid	C9H8O3	163.039	9.74	163.03903、119.04898、93.03314
38	Naringeninchalcone	C15H12O5	273.0756	9.95	273.07571、153.01837、147.04420、119.04956
39	Ferulic acid	C10H10O4	177.0547	10.234	193.05006、178.02632、149.05991、134.03621
40	Quercetin-3β-D-GLEucoside	C21H20O12	463.0887	10.733	463.08908、301.03531、300.02786
41	Quercetin	C15H10O7	303.0498	11.238	303.05051、229.04938、153.01831、137.02357
42	Trifolin	C21H20O11	447.0934	11.376	447.09567、284.03296、255.03000、227.03484
43	Astragalin	C21H20O11	447.0934	11.735	447.09711、284.03305、255.03012、227.03488
44	Kaempferol	C15H10O6	287.055	11.739	287.05481、153.01822、121.02862
45	Heptanophenone	C13H18O	191.1431	11.935	191.14317、133.10138、121.06503、69.07052
46	Dodecanedioic acid	C12H22O4	229.144	12.044	229.14465、211.13664

47	Azelaic acid	C9H16O4	187.097	12.089	187.09695 169.08598 125.09612
48	2,4-Dimethylbenzaldehyde	C9H10O	135.0806	13.063	135.0808、107.08591、105.07027、91.05473、79.05477
49	(±)-Abscisic acid	C15H20O4	263.1288	13.125	263.12924、219.13844、204.11504、201.12820
50	(prop-2-enoic acid	C11H12O4	191.0702	13.322	191.07039、163.07544、148.05197
51	(+)-ar-Turmerone	C15H20O	217.1588	15.685	217.15887、119.08578
52	Corchorifatty acid F	C18H32O5	327.2179	16.77	327.21811、229.14401、211.13405、171.10216
53	(3β,5ξ,9ξ)-3,6,19-Trihydroxyurs-12-en-28-oic acid	C30H48O5	489.3572	16.931	205.15854、201.16379、187.14810、147.11684、119.08587
54	Ganosinensic acid A	C27H38O6	459.2738	16.952	381.24222、293.15369
55	Me lucidenate N	C28H42O6	475.3053	17.077	475.26880、139.07516、69.03405
56	12β-acetoxy-3β,28-dihydroxy-7,11,15,23-tetraoxo-5α-lanosta-8-en-26-oic acid	C32H44O10	589.3018	17.202	279.07263、212.55090、153.86908、138.94522
57	13(S)-HOTrE	C18H30O3	295.227	18.01	295.22720、277.21603、95.08607、81.07040、67.05479

58	(15Z)-9,12,13-Trihydroxy-15-octadecenoic acid	C18H34O5	329.2336	18.013	329.23373、171.10197、139.11221、127.11225
59	Lucidenic acid B	C27H38O7	473.2547	18.09	150.52621、121.36600
60	(2β,3β)-3-(β-D-GLEucopyranosyloxy)-2,23-dihydroxy-30-methoxy-30-oxoolean-12-en-28-oic acid	C37H58O12	693.3864	18.349	223.64006、220.99306、166.75558、138.47670
61	12β-acetoxy-3β,7β-dihydroxy-11,15,23-trioxo-lanost-8,16-dien-26-oic acid	C32H44O9	573.3066	19.117	477.2641、459.25372、383.22202、365.21161、347.20096、329.19046
62	Arjungenin	C30H48O6	503.3385	19.33	503.33856、407.29785
63	Ganoweberianic acid F	C30H40O8	529.2795	19.653	381.20581、363.19516、345.18488、225.09091
64	2-Amino-1,3,4-octadecanetriol	C18H39NO3	318.3002	20.281	318.30023、300.28964、256.26349
65	Oleanoicacid3-O-GLEucuronide	C36H56O9	677.3913	20.966	413.0153
66	Tanariflavanone A	C30H36O7	567.2601	21.165	567.26013、549.25067、507.23947
67	Ganodernoid D	C32H40O9	569.2744	21.17	363.19626、293.15369、225.09138

68	3β-hydroxy-12β-acetoxyganodernoid D	C32H42O9	571.2899	21.441	381.20624、363.19513、345.18488、225.09082
69	(3β,5ξ,9ξ)-3,23-Dihydroxy-1-oxoolean-12-en-28-oic acid	C30H46O5	485.3277	22.53	485.3278
70	(2α,3β,19α)-2,3,19-Trihydroxyolean-12-en-28-oic acid	C30H48O5	471.3471	23.842	487.34351、469.33093、425.34470
71	(+/-)9,10-dihydroxy-12Z-octadecenoic acid	C18H34O4	313.2389	24.56	313.23895、201.11272、171.10159、127.11173、125.09579
72	12β-Acetoxy-3,7,11,15,23-pentaoxo-5α-lanosta-8-en-26-oic acid ethyl ester	C34H46O9	24.779	2415448	557.24402、225.53111、200.58018、159.10706、113.41397、67.38070、67.37757
